# Heavy Metals Removal from Aqueous Solutions by Multiwall Carbon Nanotubes: Effect of MWCNTs Dispersion

**DOI:** 10.3390/nano11082082

**Published:** 2021-08-17

**Authors:** Ana Rita Oliveira, António Alberto Correia, Maria Graça Rasteiro

**Affiliations:** 1 Department of Chemical Engineering, CIEPQPF, University of Coimbra, Rua Sílvio Lima, 3030-790 Coimbra, Portugal; anaritadeoliveira_16@hotmail.com; 2 Department of Civil Engineering, CIEPQPF, University of Coimbra, Rua Sílvio Lima, 3030-788 Coimbra, Portugal; aalberto@dec.uc.pt

**Keywords:** carbon nanotubes, heavy metal ions, surfactants, adsorption, water treatment

## Abstract

Carbon nanotubes (CNTs) are one of the most studied nanoparticles due to their physical, chemical and electronic properties. However, strong Van der Waals bonds, which promote CNTs aggregation are usually present, affecting their unique properties. Avoiding CNTs aggregation is one of the main difficulties when using these nanoparticles. Regarding the adsorption capacity of CNTs, the tendency of CNTs to aggregate decreases the surface area available to retain contaminants. One way to overcome this issue is by changing the surface energy of CNTs through chemical (covalent and noncovalent methods) or mechanical stabilization, but there is not yet a unique solution to solve this problem. In this work, a chemical noncovalent method (addition of surfactants) combined with mechanical energy (ultrasounds) was applied for CNTs stabilization, and the influence in heavy metal ions removal, Pb (II), Cu (II), Ni (II) and Zn (II), an area of high environmental relevance, was evaluated. It was proved that high amounts of metals could be removed from water during the first eighteen hours. Competitive adsorption between heavy metals, during adsorption tests with the simultaneous presence of all ions, was also studied and it was possible to prove that the electronegativity and atomic radius of cations influence their removal. Pb (II) and Cu (II) were the metals removed in higher percentages, and Ni (II) and Zn (II) were the metals less removed during competitive adsorption. Finally, the results obtained show that MWCNTs, if adequately dispersed, present a good solution for the treatment of water contaminated with highly toxic heavy metals, even when using very low concentrations of Multiwall Carbon Nanotubes (MWCNTs).

## 1. Introduction

The water crisis can become one of the most demanding problems that our society can face in the future, not only because of the high pollution levels, but also because of the very high population growth. According to the United Nations (UN), it is estimated that in 2050, Earth will have 9.3 billion inhabitants [1], corresponding to a water demand growth of 55% [2]. Thus, since water plays a pivotal role in sustainable development, which includes economic, social and environmental issues, this crisis will contribute to social inequalities, environmental imbalance and a consequent conflict between nations. It is up to governments and world organizations to control this serious problem, minimizing risks and maximizing benefits, which means implementing measures of Prevention, Reutilization and Recovery.

Moreover, as a consequence of urbanization and industrialization growth, water sources have been polluted by several substances, such as organic and inorganic pollutants, heavy metals and others, leading to an unbalanced ecosystem, changing the physical, chemical and biological state of water [3,4]. Heavy metals have raised scientific interest and strong public concern, since organisms cannot excrete these compounds, and they will accumulate in tissues and promote serious diseases, such as cancer and mutagenic problems [5,6].

For a long time, the scientific community has been studying and developing simple and advanced technologies of water treatment, based on coagulation, flocculation, filtration and disinfection, such as chemical precipitation, ion exchange, membrane filtration, adsorption and others [3,7,8]. The biggest challenge in water and wastewater treatment is the selection of the most sustainable strategy, considering the treatment cost, water quality goals, simplicity and maintenance of operations [9,10]. Nowadays, the concept of applying multiple barriers, which corresponds to combining multiple techniques in water treatment, is the basis of safe drinking water production, since it can ensure that a failure in one barrier can be compensated by the next barrier [11]. In fact, it is possible to achieve high percentages of pollution removal with this strategy (~99%) [12]. However, these techniques have been shown to require high operational costs and, in some cases, it is difficult to proceed with the scale up. On the other hand, in terms of water for human use, these techniques cannot remove or neutralize some pathogenic agents or certain compounds [12]. 

In the case of heavy metals, they are, nowadays, usually removed from wastewater applying the common techniques mentioned above, but as referred to previously, they are associated with difficult scale-up and also sometimes a high volume of toxic sludge and high maintenance cost [8,13,14,15]. Among those techniques, adsorption appears at the forefront as one of the most applied techniques in resilient substances removal, since it offers flexibility in design and operation and, in some cases, it is also possible to regenerate the adsorbent [16].

In this way, taking advantage of the carbon nanotubes (CNTs) properties to remove high percentages of resilient contaminants, such as metal ions, can become a cost-effective solution in water treatment when combined with other techniques. These nanoparticles are seen as the revolution of nanotechnologies due to their remarkable properties [17]. Besides their porous and hollow structure, contributing to a high specific surface area and a low density, with these new materials, it is possible not only to adsorb toxic substances, but also to assure the inactivity of pathogenic agents, such as bacteria and viruses [18,19,20,21]. In fact, CNTs have already been employed as a new adsorbent in heavy metal ions removal, such as Cr (VI) [22], Cu (II) [23], Ni (II) [24,25], Pb (II) [8,26] and others [27,28,29]. However, in many situations described in the literature, CNTs have to be previously activated (MWCNTs oxidation or other modifications such as grafting of amine groups, to guarantee the heavy metals removal [8,20,30]). Furthermore, there are studies reported in the literature where either ionic liquids (IL) or deep eutectic solvents (DES) are used as functionalization and/or dispersion agents in the synthesis of carbon nanotubes used in heavy metals removal, among other applications [31]. In other studies, the modification of the morphological structure of the CNTs is conducted to enhance the removal capacity of recalcitrant pollutants [32]. On the other hand, it is widely recognized that these modifications are complex [30,31], and still not at the level of a possible industrial application, also due to the costs involved. In addition, some concerns are still related to CNTs use, namely the fact that there is not a lot information about their effect on human and environmental exposure. Still, a recent study [33] has proved that, if used in low amounts, CNTs can be beneficial as a plant growth regulator in agriculture.

Moreover, their extreme tendency to form aggregates due to their hydrophobicity and high surface area can constitute a problem. This point is seen as an issue in terms of adsorption efficiency, which means that it is necessary to apply methods for stabilization of the CNTs in order to prevent CNTs aggregation [34,35,36]. There are chemical and mechanical methods to disperse CNTs suspensions. In the first case, there is a modification of surface energy, changing (covalent methods) or not (noncovalent methods) the structure of CNTs [31,37,38,39]. The use of surfactants (amphiphilic polymers) is one of the most-used chemical/noncovalent method to disperse the CNTs. However, surfactants alone cannot always promote a perfect dispersion of CNTs. In this case, applying a combination between surfactants addition and a mechanical method, such as sonication, is enough to overcome this problem most of the time, promoting good-quality dispersions with no visible aggregates [38,40,41]. Still, when using CNTs for adsorption purposes, namely for heavy metals adsorption, it is necessary to guarantee that the surfactants do not interfere with the adsorptive capacity of the CNTs.

In Continental Portugal, the main sources of heavy metal ions pollution are industrial complexes or mining exploitation [42]. According to the Annual Report of Water and Wastewater Services [43], in 2015, the metals found in larger amounts in waters for human consumption were Pb (II), Ni (II), Cu (II) and Zn (II). 

This work is focused on the application of CNTs in Pb(II), Cu (II), Ni (II) and Zn (II) removal from aqueous solutions, without making use of expensive modification strategies of the CNTs and using very low dosages of these nanoparticles. The efficiency of three different types of surfactants (anionic (SDBS), non-ionic (Pluronic F-127) and cationic (polyDADMAC MMW), was evaluated, not only in terms of the quality of CNTs dispersions, but also in terms of heavy metal ions removal efficiency, allowing us to reach conclusions about which surfactant has better performance considering the balance between a good dispersion of CNTs and the preservation of active adsorption sites. A mixture of two surfactants, SDBS and Pluronic F-127, was also tested, to evaluate if there could be complementary beneficial effects both on CNTs dispersion and heavy metal ions removal. Since, most often, several heavy metals are simultaneously present in the contaminated water, a competitive adsorption test between the four metal ions (where all metals were considered simultaneously in solution) was also performed, going beyond what has been presented in the literature, in order to conclude about their different affinity to CNTs. In this work, the quality of the CNTs dispersions was assessed by light scattering techniques, namely Dynamic Light Scattering (DLS), and the concentration of heavy metal ions in solution was measured by Atomic Absorption Spectrometry (AAS).

## 2. Materials and Methods

### 2.1. Materials

In this work, we chose to use Multiwall Carbon Nanotubes (MWCNTs) CN7000, produced by Nanocyl (Belgium), mainly due to economic factors. MWCNTs are much less expensive than Single-Wall Carbon Nanotubes (SWCNTs) and, so far, only MWCNTs are able to be produced at an industrial level. According to data provided by the supplier, these MWCNTs have a mean diameter of 9.5 nm, a mean length around 1500 nm and a specific surface area between 250,000 and 300,000 m^2^/kg. They are composed of 90% (*w*/*w*) of pure carbon and 10% (*w*/*w*) of metallic oxides. Further tests completed the characterization of the MWCNTs supplying information about density (1.7 g/cm^3^) and zeta potential at neutral pH (−25.2 mV) [44,45].

In terms of surfactants, three different types, provided by Sigma-Aldrich (St. Louis, MO, USA), were tested: The anionic surfactant SDBS, the non-ionic Pluronic F-127 and a cationic surfactant polyDADMAC MMW. The reasoning behind this choice was to check both the effect of charge and molecular weight of the surfactant on the ability to disperse the MWCNTs. It is important to notice that the surfactants’ characterization was available on data provided by the supplier and was completed by tests performed previously at the Chemical Engineering Department of the University of Coimbra [46] (Table 1). 

Heavy metal ion solutions were prepared dissolving the respective salts with deionized water. In this way, solutions of copper (II) chloride dehydrated salt, CuCl_2_·2H_2_O, nickel (II) sulfate hexahydrated, Ni(SO_4_)·6H_2_O, lead (II) nitrate, Pb(NO_3_)_2_ and zinc sulphate heptahydrated, ZnSO_4_·7H_2_O, were prepared. These salts were supplied by Merck (Darmstadt, Germany), Fluka AG (Buchs, Switzerland), Riedel (Seelze, Germany) and Sigma-Aldrich (St. Louis, MO, USA), respectively.

### 2.2. Experimental Procedures

This work is based on two experimental procedures. First, the preparation of the MWCNTs suspensions in the surfactants solutions. Second, the preparation of heavy metal ions solutions. At the end, the better-quality dispersions were added to the metal ions solutions and left under stirring conditions for 7 days, and adsorption tests were conducted.

#### 2.2.1. Preparation of MWCNTs Suspensions

Firstly, the MWCNTs dispersions were prepared applying ultrasonic energy, using an ice bath with water flux to control the temperature. The optimal conditions of sonication were defined previously, on preliminary tests, as 15 min of sonication time and 75% of sonication energy (375 W). The sonication equipment used was a probe-sonicator from Sonics, model Vibracell 505 (Newtown, CT, USA) with a frequency of 20 kHz and power of 500 W.

For each surfactant, 150 mL of solution was prepared using deionized water. The optimal concentration of the surfactant determined previously (leading to the best CNTs dispersion) was 0.03% (*w*/*w*) for each surfactant. The surfactant solution was prepared by stirring during 12 h at 500 rpm. In the case of the mixture of surfactants, 0.03% (*w*/*w*) of SDBS and 0.03% (*w*/*w*) of Pluronic F-127 were used, for a volume of 150 mL of solution. 

MWCNTs were weighted in order to obtain a concentration of 0.01% (*w*/*w*) in the suspension, corresponding to 0.015 g of MWCNTs for a volume of surfactant solution equal to 150 mL. 

After the surfactant solution had been homogenized, the MWCNTs were added and the suspension submitted to sonication energy under the conditions mentioned above. The quality of the dispersions was controlled using the DLS technique (Dynamic Light Scattering), using a Zetasizer nanoZS, Malvern, UK, which supplies the particle size distribution of the MWCNTs in the suspension.

#### 2.2.2. Preparation of Heavy Metal Ions Solutions

Heavy metal ion solutions were prepared as follows: Each salt corresponding to each metal was weighted, added to four different vessels filled with deionized water and left under stirring conditions during 30 min at 500 rpm. These solutions were diluted several times in order to obtain four single solutions with, respectively, ~3 mg/L of Pb (II), ~5 mg/L of Cu (II), ~3 mg/L of Ni (II) and ~3 mg/L of Zn (II) and left again under stirring conditions during 10 min at 500 rpm. From these solutions, 50 mL were taken and added to four different vessels, containing 150 mL of the previously prepared MWCNTs suspensions, already dispersed, with the purpose to treat the contaminated water with the MWCNTs. The exact concentration of each heavy metal ion in the suspension of MWCNTs will be presented in the tables in the next section, for the time equal to zero. In another test, the same concentration of Pb (II) was treated with a mixture of two surfactants.

For the competitive adsorption test, between all the heavy metal ions, the salts were weighted and added to a single vessel filled with deionized water and left under stirring conditions as already described. This solution was diluted several times in order to obtain a solution with ~4 mg/L of Pb (II), ~7 mg/L of Cu (II), ~3 mg/L of Ni (II) and ~3 mg/L of Zn (II) and left under stirring conditions as already described. From this solution, 50 mL were taken to add to a vessel containing 150 mL of the MWCNTs suspension already dispersed, as described for the single metals tests. Again, the exact concentration of each heavy metal ion in the MWCNTs suspension will be presented in the tables in the next section, for the time equal to zero.

#### 2.2.3. Adsorption Tests

The different mixtures of aqueous suspensions containing heavy metals and dispersed MWCNTs were maintained under stirring conditions for 7 days at 100 rpm. During the tests, samples of 10 mL were taken after 4 h, 18 h, 24 h and 7 days. Those samples were collected and filtered, first with a membrane filter of 1.0 μm and then filtered again with a membrane filter of 0.45 μm. The number of cations in the filtered water was quantified by AAS, Atomic Absorption Spectrometer Perkin Elmer 3300 (Perkin Elmer, Waltham, MA, USA), and the amount of adsorbed metal was calculated by comparison with the initial number of cations in the solution. All tests were performed in duplicate.

## 3. Results and Discussion

### 3.1. MWCNTs Suspensions

**Figure 1:**
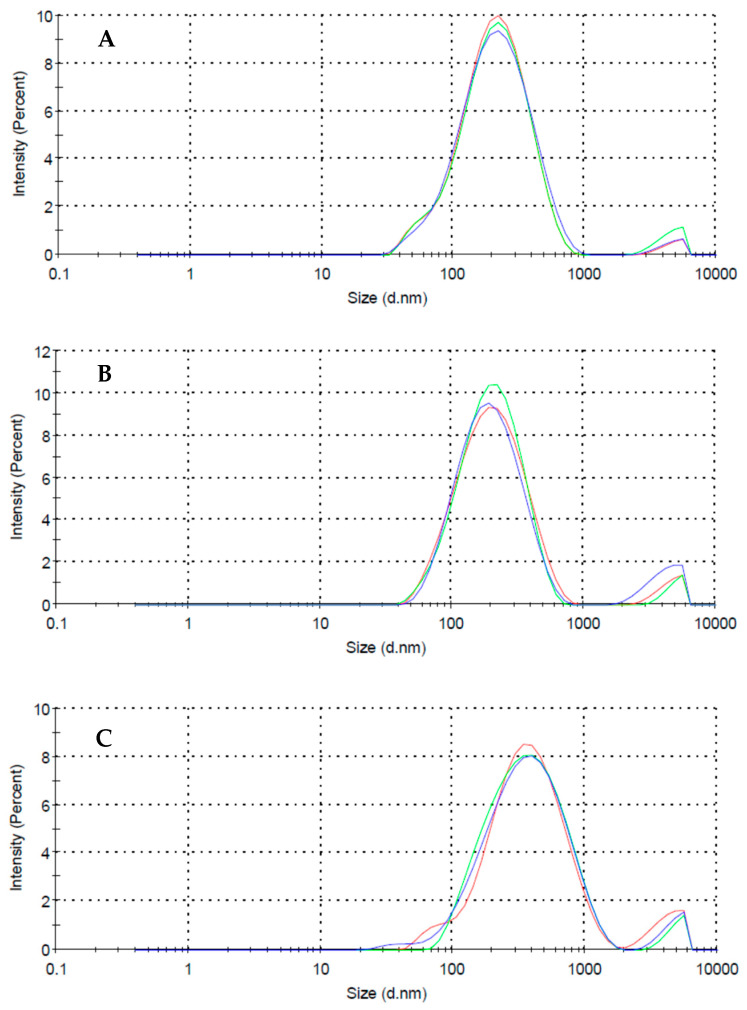
Intensity-weighted particle size distribution of the particles in a dispersion of 0.01% (*w*/*w*) of MWCNTs applying SDBS (**A**), Pluronic F-127 (**B**) and polyDADMAC MMW (**C**) as surfactant. Concentration of surfactant equal to 0.03% (*w*/*w*).

The quality of MWCNTs dispersions obtained by DLS is analyzed based on the value of the D_z_ average diameter obtained for each suspension (Table 2). First, the possibility of dispersing the MWCNTs applying only ultra-sounds and no surfactant was evaluated, with the existence of several aggregates being visible to the naked eye, and the average diameter of the MWCNTs size distribution being 536.4 nm. When applying SDBS and Pluronic F-127, the average diameter obtained was 180.5 nm and 187.3 nm, respectively. This means that good-quality dispersions were reached. When a concentration of 0.03% (*w*/*w*) of polyDADMAC MMW was used, it was not possible to completely overcome the problem of MWCNTs aggregation. The average diameter in this case was equal to 307.7 nm and some MWCNTs aggregates were visible (Figure 1c). Increasing the concentration of polyDADMAC MMW did not improve the quality of the dispersion. Even with this problem, adsorption tests were also performed with this surfactant for comparison purposes. Figure 1 shows the size distribution of the suspensions of 0.01% (*w*/*w*) MWCNTs with 0.03% (*w*/*w*) of SDBS, Pluronic F-127 and polyDADMAC MMW. It is possible to observe that in the case of polyDADMAC MMW, the size distribution is wider and positioned more to the right (larger diameters) compared with the other two distributions. 

Although SDBS has a negative zeta potential (as do the MWCNTs), a good-quality dispersion was reached, which is explained by the adsorption of the hydrophobic surfactant tail on the nanotubes. On the other hand, for Pluronic F-127 and polyDADMAC MMW, the binding between these polymers and MWCNTs surface was probably based on Van der Waals and Coulomb electrostatic forces, the latter leading to a worse dispersion of the MWCNTs.

### 3.2. Heavy Metal Ions Removal

**Figure 2:**
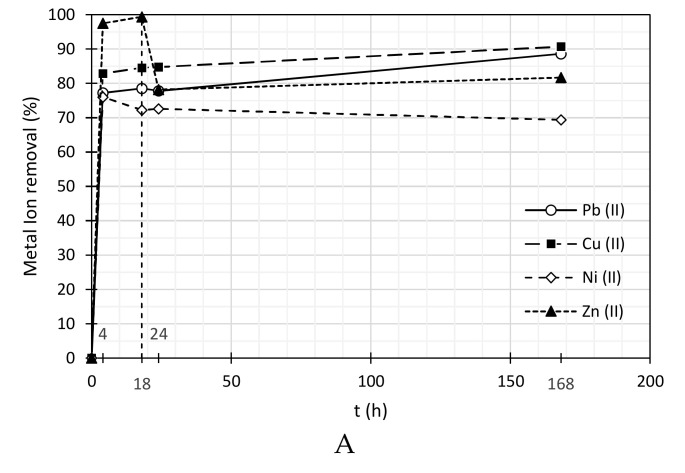

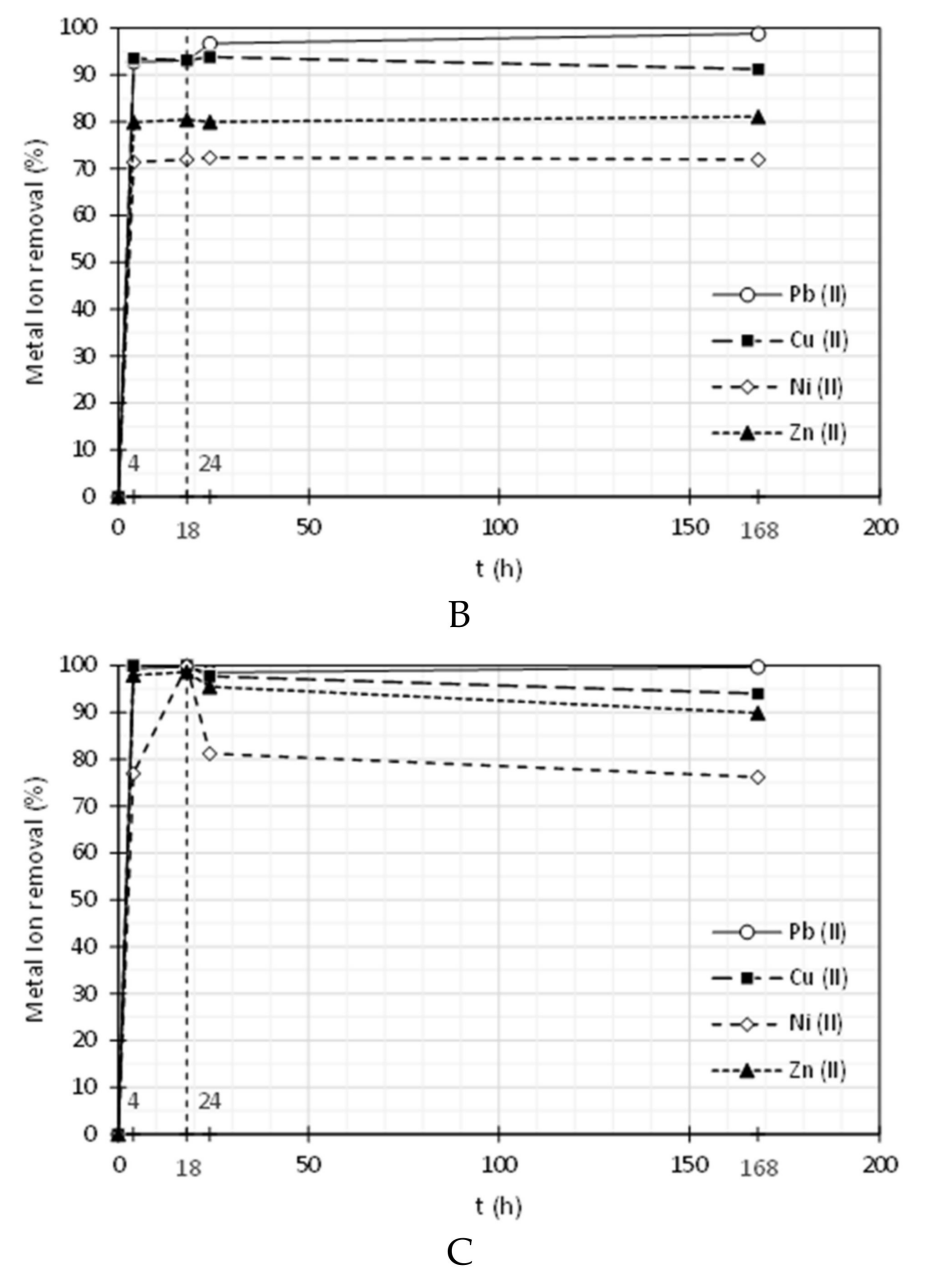
Metal ion removal by suspensions of 0.01% (*w*/*w*) of MWCNTs dispersed by SDBS (**A**), Pluronic F-127 (**B**) and polyDADMAC MMW (**C**). All surfactants concentrations were equal to 0.03% (*w*/*w*).

Adsorption tests were performed only for situations where a good dispersion of the MWCNTs could be obtained, that is, applying both ultrasounds and the selected surfactants. In general, it was possible to reach heavy metal ions removal above 70% (Table 3, Table 4 and Table 5). Paying attention to Figure 2, tests performed with Pluronic F-127 show the highest removal for all the cations. It is also possible to conclude that Pb (II) and Cu (II) were the metals with higher percentage removal and Ni (II) and Zn (II) were the metals less removed, after 7 days of adsorption. Additionally, removal was always faster when using Pluronic F-127. PolyDADMAC MMW shows better removal than SDBS for Pb (II) and similar removals for Cu (II), Zn (II) and Ni (II), after 7 days. The better removal when Pluronic F-127 is used as surfactant may be related to its much smaller hydrodynamic diameter (Table 1) which, nevertheless, still leads to a very good dispersion of the MWCNTs. Pluronic F-127 allows, on one hand, a good dispersion of the MWCNTs (Table 2) and, on the other hand, its molecules do not occupy a large percentage of the MWCNTs surface, due to its smaller size, leaving a larger percentage of the carbon nanotubes surface free for the heavy metals adsorption.

In terms of Pb (II), it was possible to reduce its initial concentration (~3 mg/L) to 0.34 mg/L (88.6% of removal) using SDBS, 0.04 mg/L (98.8% removal) for polyDADMAC MMW and 0.01 mg/L (99.7% removal) for tests performed with Pluronic F-127. In the case of Cu (II) tests, it was possible to decrease its initial concentration to 0.22 mg/L (90.7% removal), 0.47 mg/L (91.3% removal) and 0.32 mg/L (94.0% removal) applying SDBS, polyDADMAC MMW and Pluronic F-127, respectively. For Zn (II), its initial concentration was decreased to 0.63 mg/L (81.7% removal), 0.72 mg/L (81.1% removal) and 0.35 mg/L (89.9% removal) when applying SDBS, polyDADMAC MMW and Pluronic F-127, respectively. Finally, in terms of Ni (II) adsorption tests, it was possible to reduce its initial concentration to 0.81 mg/L (69.4% removal), 1.05 mg/L (72.0% removal) and 0.63 mg/L (76.2% removal), applying the same surfactant order. In the case of the tests with polyDADMAC MMW, the slightly better removal obtained with this surfactant when compared with the results obtained with SDBS, which induces a better dispersion of the MWCNTs, may be due to the larger hydrodynamic diameter of the latter surfactant (Table 1), which can hinder the adsorption of the metal ions.

Comparing all results in Table 3, Table 4 and Table 5, the highest tendency for ions removal is for Pb (II) and Cu (II), due to their higher affinity with active adsorption sites on MWCNTs. This fact might be explained by the higher electronegativity of these cations. Pb (II) is a cation with an electronegativity around 2.33 on the Pauling scale [47], which means that it has more tendency to be removed than the other metals. Regarding electronegativity, Pb (II) is followed by Cu (II), Ni (II) and Zn (II), with values of electronegativity of 1.91, 1.90 and 1.65, respectively [45]. The difference in removal of Ni (II) and Cu (II), with similar electronegativity, may be attributed to the higher atomic radius of Ni (II) (149 pm for Ni (II) and 128 pm for Cu (II)), [45]. This effect was studied in more detail on the competitive adsorption tests between all cations, presented later in this work. The lower removal of Zn (II) may be attributed to its higher mobility due to its lower atomic radius (134 pm).

**Figure 3:**
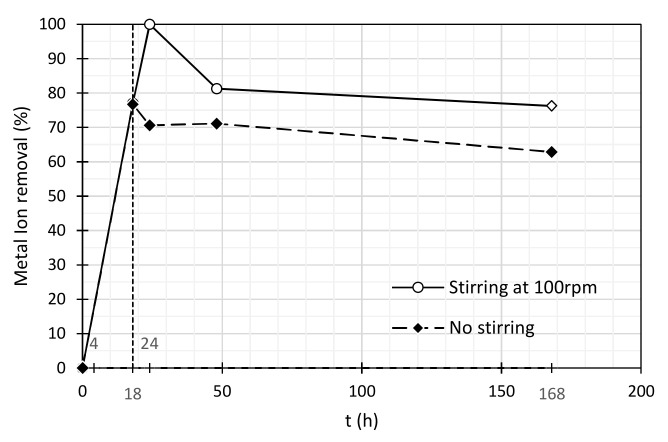
Ni (II) removal applying a suspension of 0.01% (*w*/*w*) of MWCNTs with 0.03% (*w*/*w*) of Pluronic F-127, with stirring at 100 rpm and no stirring. Initial concentration of Ni (II) ~3 mg/L.

Additionally, it is important to mention that at 18 h, some heavy metals reached their maximum percentage removal (Ni and Zn for tests with SDBS and Pluronic F-127 surfactants); however, after this time, a desorption of these cations was observed, which stabilized after some time. Hydrodynamic forces promoted by stirring conditions might explain this desorption. In this way, a test with no continuous stirring was performed, applying Pluronic F-127 as surfactant to disperse a suspension containing 0.01% (*w*/*w*) of MWCNTs and using Ni (II) as the heavy metal. Table 6 and Figure 3 show the results that confirm the explanation proposed above.

In the test with no continuous stirring, almost no fluctuations with time, on Ni (II) concentration, were observed after the occurrence of adsorption. In terms of percentage removal, if we compare both adsorption tests, it is possible to conclude that, nevertheless, stirring leads to higher percentages of removal. However, when stirring is applied, Ni(II) ions can be more easily released from the adsorption matrix, due to Brownian movements, considering the low electronegativity of this cation. This means that it is essential to optimize stirring conditions in terms of velocity and time, in order to promote a high heavy metal removal but avoiding desorption problems.

In Table 7, the results obtained in the present study, regarding the removal of heavy metals from water, using MWCNTs additives with the aforementioned surfactants, are compared with results from the literature, for removal of the same heavy metals, namely using functionalized CNTs.

It is obvious from Table 7 that the results obtained in the present study agree well with results obtained using more complex modification strategies. 

### 3.3. Competitive Adsorption Tests

**Figure 4:**
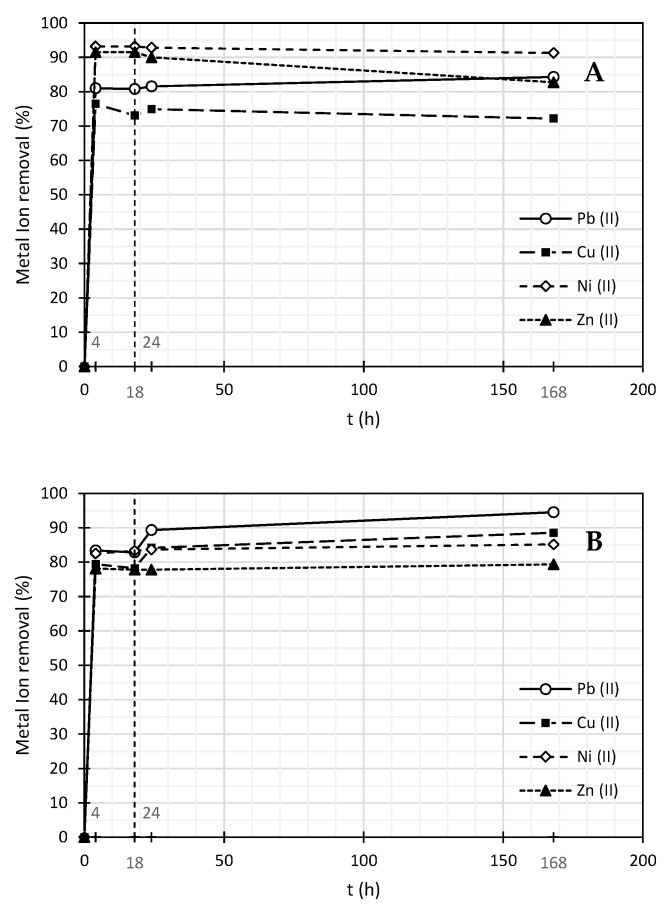
Metal ions removal by adsorption in suspensions with 0.01% (*w*/*w*) of MWCNTs and 0.03% (*w*/*w*) of SDBS (**A**), 0.03% (*w*/*w*) of Pluronic F-127 (**B**), under competitive conditions between cations. Mixture of heavy metals: Initial concentrations Pb (II) ~4 mg/L, Cu (II) ~7 mg/L, Ni (II) ~3 mg/L and Zn (II) ~3 mg/L.

The results of the competitive adsorption tests, with all heavy metals in solution at the same time, are presented in Table 8 and Table 9 and Figure 4. Tests performed with Pluronic F-127 as surfactant showed the following order of removal Pb (II) > Cu (II) > Ni (II) > Zn (II). This result comes in agreement with the single tests results, where Pb (II) showed to be more easily removed than Ni (II) or Zn (II). As already mentioned, this sequence can be explained by the electronegativity of the cations. Since Pb (II) is the heavy metal ion that is more electronegative, its tendency to be attracted and remain adsorbed on the MWCNTs structure is higher compared with the other cations. 

On the other hand, tests performed with SDBS appeared to follow a different order of removal of Ni (II) > Pb (II) > Zn (II) > Cu (II). In this case, the results may be explained not only by the electronegativity of the cations but especially by the relation between the cations atomic radius. Once SDBS has higher dimensions (hydrodynamic diameter) than Pluronic F-127, it can be a larger obstacle for bigger cations to reach the MWCNTs adsorption sites. This means that Ni (II) will reach the MWCNTs structure more easily than Pb (II), once the first one has a smaller atomic radius (149 pm compared with 175 pm of Pb (II)) [47]. So, adsorption will be controlled by the balance between electronegativity and the atomic radius of cations. In line with this, Pb (II) removal is higher than for Zn (II), which is the result of the much higher electronegativity of Pb (II) and not only of the atomic radius sequence (Pb (II) = 175 pm > Ni (II) = 149 pm > Zn (II) = 134 pm > Cu (II) = 128 pm) [47]. Furthermore, Pluronic F-127 shows a better removal percentage in the case of Pb (II), the metal cation with the highest atomic radius, since, due to its lower hydrodynamic diameter, this surfactant leaves more free sites for this larger cation adsorption on the MWCNTs surface.

Additionally, it must be stressed that even if slightly lower removals are obtained in the case of the competitive adsorption tests, when compared with the single adsorption ones, since the concentration of ions in the medium increased for the same amount of active adsorption sites on MWCNTs, with all cations competing for the same sites, removals are still quite high in the case of competitive adsorption (above 78%), which confirms the high adsorption efficiency of the MWCNTs.

### 3.4. Adsorption Tests Using a Mixture of SDBS and Pluronic F-127

The possibility of using a mixture of surfactants to disperse the carbon nanotubes was also evaluated. The mixture of surfactants seemed to be able to disaggregate the MWCNTs but with no significant difference compared with single surfactant solutions. This suspension reached an average particle diameter of 179.1 nm, which is quite similar to the situations where single SDBS or Pluronic F-127 were used, at 180.5 nm and 187.3 nm, respectively (Table 2). 

**Figure 5:**
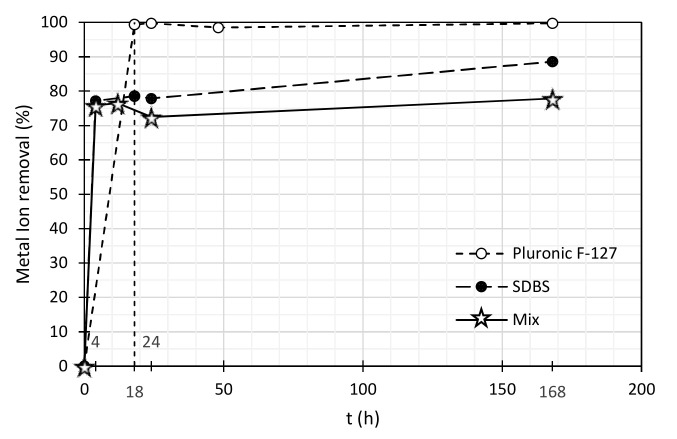
Pb(II) removal applying a suspension of 0.01% MWCNTs dispersed by a mixture of 127. 0.03% (*w*/*w*) SDBS and 0.03% (*w*/*w*) of Pluronic F-127. Pb(II) initial concentration was ~3 mg//L.

Only the removal tests for Pb (II) using the mixture of surfactants were performed. Table 10 and Figure 5 summarize the Pb (II) removal by MWCNTs, comparing the effect of the addition of the three surfactant solutions studied. It is possible to conclude that there is no significant effect of applying a mixture of surfactants in Pb (II) removal for this MWCNTs concentration. Pluronic F-127 reached a better percentage removal (99.7%) followed by SDBS (88.6%) and the mixture of these two (77.9%). A possible explanation for these results can be attributed to the surface area of the MWCNTs that remains available to receive the metal ions after the surfactants addition, since, in fact, the concentration of the surfactants was doubled when using the two surfactants simultaneously, thus covering a larger area of the MWCNTs surface.

Pluronic F-127 is a smaller molecule (hydrodynamic diameter 6.9 nm), while still promoting a good dispersion of the nanotubes, and, in this way, a better heavy metal ion removal is obtained, since it leaves more active sites for adsorption. It was expected that the mixture with SDBS, which has a negative zeta potential (−67 mV), would enhance the “trapping” effect and promote better Pb (II) removal. However, this mechanism was not dominant, probably due to the lower surface area available for adsorption when the two surfactants were used with a total double concentration.

In this way, there must be a compromise between the surfactants concentration and size and the active sites left on the MWCNTs surface for metals adsorption. It is therefore important to optimize the concentration of the surfactants in order to maximize the quality of the dispersion and the free surface area available for adsorption, to get the most out of the heavy metals’ cations removal process.

## 4. Conclusions

SDBS and Pluronic F-127 led to good results in terms of MWCNTs dispersion, contrary to polyDADMAC MMW that could not overcome completely the MWCNTs aggregation problem.

Overall, MWCNTs proved to be an efficient solution for heavy metal cations removal from wastewaters. Moreover, in terms of heavy metal ions removal, all surfactants appeared to be efficient, in spite of the slightly worst dispersion obtained with PolyDADMAC MMW. Thus, there is a good indication of the possibility of this strategy to be used in real cases of water treatment.

Pluronic F-127 combined with MWCNTs was revealed to be the best combination for cations removal, removing 99% of Pb (II), 94% of Cu (II), 90% of Zn (II) and 76% of Ni (II) from the aqueous systems. However, after 18h of testing, a significant desorption of Ni (II) and Zn (II) was registered, which is explained by the hydrodynamic forces provided by stirring conditions. This phenomenon was proved by conducting studies with and without stirring. However, it must be taken into consideration that without stirring, the overall metal cations removal could not be improved, even if the desorption phenomenon did not occur. This leads to the conclusion that adsorption time and stirring conditions need to be optimized, in order to avoid the later release of cations and maximize adsorption, especially in real water treatments.

SDBS worked as a “trap” to cations due to its anionic nature. However, this effect was not totally significant in terms of cations adsorption due to the higher hydrodynamic diameter of this surfactant, which leads to the reduction of the adsorption sites available in the MWCNTs surface. Again, to maximize removal, it is necessary to optimize the balance between the dispersion ability of the surfactant and the existence of enough adsorption sites on the MWCNTs surface, not occupied by the surfactant.

In competitive tests, SDBS was only more effective in the case of Ni (II) and Zn (II). In this situation, the atomic radius of the cations can explain why Ni (II) and Zn (II), cations with less electronegativity but with a smaller atomic radius, which, in principle would be more difficult to trap, showed a higher affinity with the MWCNTs surface, when anionic SDBS was used as surfactant. Still, in terms of competition between heavy metal ions, Pluronic F-127’s effectiveness was in agreement with the electronegativity of the Pauling scale, once Pb (II) was the cation more attracted to the complex surfactant/MWCNTs, followed by Cu (II), Ni (II) and Zn (II).

In general terms, the better efficiency on cations removal achieved when Pluronic F-127 was used as surfactant can be related to the smaller size of this molecule, which, while allowing a good dispersion of the MWCNTs, leaves more free active sites for the heavy metal cations adsorption on the carbon nanotubes surface.

To summarize, the importance of optimizing the surfactants concentration and type must be stressed, keeping in mind the balance between MWCNTs dispersion and surfactants molecules size that conditions the free active surface on the MWCNTs, which is left available for the adsorption process. Finally, if a good dispersion of the MWCNTs is guaranteed, this nanomaterial proved to be an efficient solution for the removal of heavy metals from contaminated waters, even when using a very low concentration of MWCNTs (0.01% *w*/*w*). In the future, it will be important to develop matrixes to incorporate and fix the MWCNTs to facilitate the introduction and use of this strategy in real wastewater treatment systems and to increase the capacity of re-utilization of the CNTs. Moreover, an evaluation of the eventual toxicity of the MWCNTs, if released in the environment, will also be a focus for future studies.

## Figures and Tables

**Table 1:** Summary of surfactants characterization [46].

Surfactant	Charge (−)	Chain Type	D_z_^av^ (nm)	Z^av^ (mV)	MW^av^ (kDa)
SDBS	Anionic	Linear	81.02	−66.97	363
Pluronic F-127	Non-ionic	Linear	6.92	−0.43	9.49
PolyDADMAC MMW	Cationic	Linear	48.58	69.47	240

**D_z_^av^**—z- Average Hydrodynamic Diameter (determined by DLS—Dynamic Light Scattering); **Z^av^**—Average Zeta Potential (determined by ELS—Electrophoretic Light Scattering); **MW^av^**—Average Molecular Weight (determined by SLS—Static Light Scattering).

**Table 2:** Summary of Z-average diameter for MWCNTs dispersions with concentrations of 0.01% (*w*/*w*) applying different surfactants.

Surfactant	Surfactant Concentration (*w*/*w* %)	MWCNTs Concentration (*w*/*w* %)	D_z_^av^ (nm)
No-surfactant	-		536.4 ± 4.5
SDBS	0.03	0.01	180.5 ± 2.3
Pluronic F-127	0.03		187.3 ± 3.8
PolyDADMAC MMW	0.03		307.7 ± 6.3
	0.1		363.0 ± 23.1

**Table 3:** Removal of Pb (II), Cu (II), Ni (II) and Zn (II) by a suspension of 0.01% (*w*/*w*) of MWCNTs in a solution of 0.03% (*w*/*w*) of SDBS.

SDBS								
	Pb (II)		Cu (II)		Ni (II)		Zn (II)	
Time (h)	Concentration (mg/L)	% of Removal	Concentration (mg/L)	% of Removal	Concentration (mg/L)	% of Removal	Concentration (mg/L)	% of Removal
0	2.98	0.0	2.38	0.0	2.64	0.0	3.43	0.0
4	0.68	77.2	0.41	82.8	0.63	76.0	0.09	97.5
18	0.64	78.5	0.37	84.6	0.73	72.2	0.02	99.4
24	0.66	77.9	0.36	84.8	0.72	72.6	0.75	78.2
168	0.34	88.6	0.22	90.7	0.81	69.4	0.63	81.7

**Table 4:** Removal of Pb(II), Cu (II), Ni (II) and Zn (II) by a suspension of 0.01% (*w*/*w*) of MWCNTs in a solution of 0.03% (*w*/*w*) of Pluronic F-127.

Pluronic F-127								
	Pb (II)		Cu (II)		Ni (II)		Zn (II)	
Time (h)	Concentration (mg/L)	% of Removal	Concentration (mg/L)	% of Removal	Concentration (mg/L)	% of Removal	Concentration (mg/L)	% of Removal
0	3.33	0.00	5.41	0.00	2.64	0.00	3.43	0.00
4	0.02	99.4	0.00	100	0.61	77.0	0.07	98.0
18	0.01	99.7	0.00	100	0.00	100	0.05	98.6
24	0.05	98.5	0.12	97.8	0.50	81.2	0.15	95.5
168	0.01	99.7	0.32	94.0	0.63	76.2	0.35	89.9

**Table 5:** Removal of Pb(II), Cu (II), Ni (II) and Zn (II) by a suspension of 0.01% (*w*/*w*) of MWCNTs in a solution of 0.03% (*w*/*w*) of polyDADMAC MMW.

PolyDADMAC MMW								
	Pb (II)		Cu (II)		Ni (II)		Zn (II)	
Time (h)	Concentration (mg/L)	% of Removal	Concentration (mg/L)	% of Removal	Concentration (mg/L)	% of Removal	Concentration (mg/L)	% of Removal
0	3.33	0.0	5.41	0.0	3.74	0.0	3.8	0.0
4	0.24	92.8	0.35	93.6	1.07	71.4	0.76	79.9
18	0.23	93.1	0.37	93.2	1.05	71.9	0.74	80.5
24	0.11	96.7	0.33	93.9	1.03	72.4	0.76	80.0
168	0.04	98.8	0.47	91.3	1.05	72.0	0.72	81.1

**Table 6:** Ni (II) removal by a suspension of 0.01% (*w*/*w*) of MWCNTs with 0.03% (*w*/*w*) of Pluronic F-127, applying stirring at 100 rpm and with no stirring. Initial concentration of heavy metal ion ~3 mg/L.

	Stirring at 100 rpm		No Stirring	
Time (h)	Ni(II) Concentration (mg/L)	Ni (II) Removal (%)	Ni(II) Concentration (mg/L)	Ni (II) Removal (%)
0	2.64	0.0	2.64	0.0
4	0.61	77.0	0.62	76.7
18	0.0	100	0.78	70.6
24	0.50	81.2	0.76	71.1
168	0.63	76.2	0.98	62.9

**Table 7:** Comparison of removal efficiencies obtained in the present study with information from the literature.

Metal	Removal %	Reference
Ni (II)	35 76.12 92.79 100 90	Oxidized CNTs [24] Purified CNTs (HNO_3_/H_2_SO_4_, 1:3) [48] PEG modified CNTs [48] Present work—maximum removal obtained Chitosan modified CNTs [49]
Pb (II)	90.7 97.16 99.7	Purified CNTs (HNO_3_/H_2_SO_4_, 1:3) [48] PEG modified CNTs [48] Present work—maximum removal obtained
Cu (II)	99.09 99.99 95 100	Purified CNTs (HNO_3_/H_2_SO_4_, 1:3) [48] PEG modified CNTs [48] Chitosan modified CNTs [49] Present work—maximum removal obtained
Zn (II)	95.4 90.3 98.6	Oxidized CNTs, pH 5.4 [23] NaOCl purified CNTs [27] Present work—maximum removal obtained

**Table 8:** Removal of Pb(II), Cu (II), Ni (II) and Zn (II) by a suspension of 0.01% (*w*/*w*) of MWCNTs with 0.03% (*w*/*w*) of SDBS, under competitive adsorption conditions between cations.

SDBS								
**Time (h)**	Pb (mg/L)	Cu (mg/L)	Ni (mg/L)	Zn (mg/L)	% Removal			
					Pb (%)	Cu (%)	Ni (%)	Zn (%)
0	3.69	6.58	2.64	3.30	0.00	0.00	0.00	0.00
4	0.70	1.55	0.18	0.28	81.0	76.4	93.2	91.5
18	0.71	1.77	0.18	0.28	80.8	73.1	93.2	91.5
24	0.68	1.65	0.19	0.33	81.6	74.9	92.8	90.0
168	0.58	1.83	0.23	0.57	84.3	72.2	91.3	82.7

**Table 9:** Removal of Pb(II), Cu (II), Ni (II) and Zn (II) by a suspension of 0.01% (*w*/*w*) of MWCNTs with 0.03% (*w*/*w*) of Pluronic F-127, under competitive adsorption conditions between cations.

Pluronic F-127								
Time (h)	Pb (mg/L)	Cu (mg/L)	Ni (mg/L)	Zn (mg/L)	% Removal			
					Pb (%)	Cu (%)	Ni (%)	Zn (%)
0	3.69	6.58	2.64	3.30	0.00	0.00	0.00	0.00
4	0.61	1.35	0.46	0.72	83.5	79.5	82.6	78.2
18	0.63	1.43	0.44	0.73	82.9	78.3	83.3	77.9
24	0.39	1.04	0.43	0.73	89.4	84.2	83.7	77.9
168	0.20	0.75	0.39	0.68	94.6	88.6	85.2	79.4

**Table 10:** Pb (II) removal applying a suspension of 0.01% (*w*/*w*) of MWCNTs dispersed by a mixture of SDBS and Pluronic F-127, 0.03% (*w*/*w*) of SDBS and 0.03% (*w*/*w*) of Pluronic F-127.

	Pb (II) Concentration (mg/L)			Pb (II) Removal (%)		
Time (h)	Mixture of Surfactants	SDBS	Pluronic F-127	Mixture of Surfactants	SDBS	Pluronic F-127
0	2.98	2.98	3.33	0.0	0.0	0.00
4	0.72	0.68	0.02	75.8	77.2	99.4
18	0.70	0.64	0.01	76.5	78.5	99.7
24	0.82	0.66	0.05	72.5	77.9	98.5
168	0.66	0.34	0.01	77.9	88.6	99.7
